# Single-port laparoscopic-assisted resection for a large abdominal cystic lymphangioma: a case report

**DOI:** 10.1186/s40792-018-0501-9

**Published:** 2018-08-13

**Authors:** Hideki Kogo, Satoshi Matsumoto, Eiji Uchida

**Affiliations:** 1 Department of Gastrointestinal and Hepato–Biliary–Pancreatic Surgery, Nippon Medical School, 1-1-5 Sendagi, Bunkyo-ku, Tokyo, 113-8602 Japan; 2 Department of Surgery, Nippon Medical School Chiba Hokusoh Hospital, Chiba, Japan

**Keywords:** Large abdominal cystic lymphangioma, Single-port laparoscopic resection, Mesenteric cyst

## Abstract

**Background:**

We report the case of a young woman with a large abdominal cystic lymphangioma that was successfully resected using single-port laparoscopic-assisted cystectomy. This avoided the need for a large surgical incision, as would result during conventional laparotomy.

**Case presentation:**

A 17-year-old young woman was admitted to our hospital complaining of abdominal pain that had persisted for 3 days. Computed tomography revealed a 10 × 10 × 10-cm low-density area in the mid-abdomen, and magnetic resonance imaging showed a large abdominal cystic lesion. A mesenteric cyst was suspected, and single-port laparoscopic-assisted resection was performed. The cyst fluid was aspirated using a tissue adhesive, a suction tube with negative pressure, and a 16-gage over-the-needle catheter and syringe. The tumor size was reduced without any spillage of cyst fluid into the abdominal cavity. Then, the shrunken cystic tumor was successfully removed via the small wound and resected outside the abdomen. Pathological findings revealed an abdominal cystic lymphangioma derived from the greater omentum.

**Conclusions:**

Our procedure was easy to perform and required no special materials. Therefore, it could be applied to various cases, such as for abdominal cystic diseases.

## Background

Lymphangiomas are isolated tumors of lymphatic vessels that have become disconnected from the normal lymphatic system [1–3]. Around 65% of cystic lymphangiomas occur at birth, with 90% occurring in patients aged < 2 years; 75% are found in the neck and 20% in the axilla [3], with fewer than 5% being abdominal. They present in the mesentery in approximately 1% of cases, and within this group, they are most commonly found in the small bowel (85%), mesocolon (10%), and retroperitoneum (5%) [3]. Abdominal lymphangiomas are often asymptomatic and can be accompanied by symptoms of compression of the neighboring organs resulting from the growth of the mass [4]. It is possible for bleeding or infection to result in acute abdominal disease [5, 6]. Lymphangioma is included in the differential diagnosis of malignant tumors such as sarcoma and cystadenocarcinoma [7].

The content of the cyst may be infected; therefore, the tumor must be resected without the spillage of any cyst fluid into the abdominal cavity. In this report, we describe a case of a rare abdominal cystic lymphangioma, which we successfully resected using single-port laparoscopic-assisted cystectomy. This avoided the need for a large surgical incision as required by conventional laparotomy.

## Case presentation

A 17-year-old woman was admitted to our hospital complaining of abdominal pain that had persisted for 3 days. She seemed alert and was not pale, with blood pressure of 112/70 mmHg and a regular pulse of 78 bpm. Laboratory data showed a white blood cell count of 7530/μL, hemoglobin concentration of 11.0 g/dL, a platelet count of 249,000/μL, glutamic oxaloacetic transaminase concentration of 22 IU/L, glutamic pyruvic transaminase concentration of 9 IU/L, and lactic dehydrogenase concentration of 259 IU/L.

**Fig. 1:**
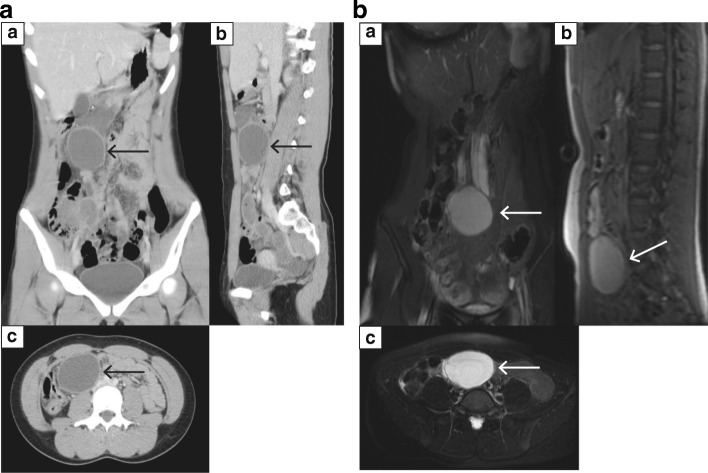
Imaging studies. **a** Computed tomography of the abdomen. Coronal (a), sagittal (b), and axial (c) images show a well-defined, rounded mass measuring 10 cm along the greatest dimension (arrows). **b** T2-weighted fast spin echo magnetic resonance image of the abdomen. Coronal (a), sagittal (b), and axial (c) images show a well-defined, rounded mass measuring 10 cm along the greatest dimension (arrows), indicative of a mesenteric cyst

Computed tomography (CT) revealed a 10 × 10 × 10-cm low-density area in the patient’s mid-abdomen (Fig. 1a), and magnetic resonance imaging (MRI) showed a large abdominal cystic lesion (Fig. 1b). However, the tumor position differed notably between CT and MRI, and an unfixed, mesenteric cystic lesion was suspected. Single-port laparoscopic-assisted resection was therefore performed instead of conventional laparotomy.

**Fig. 2:**
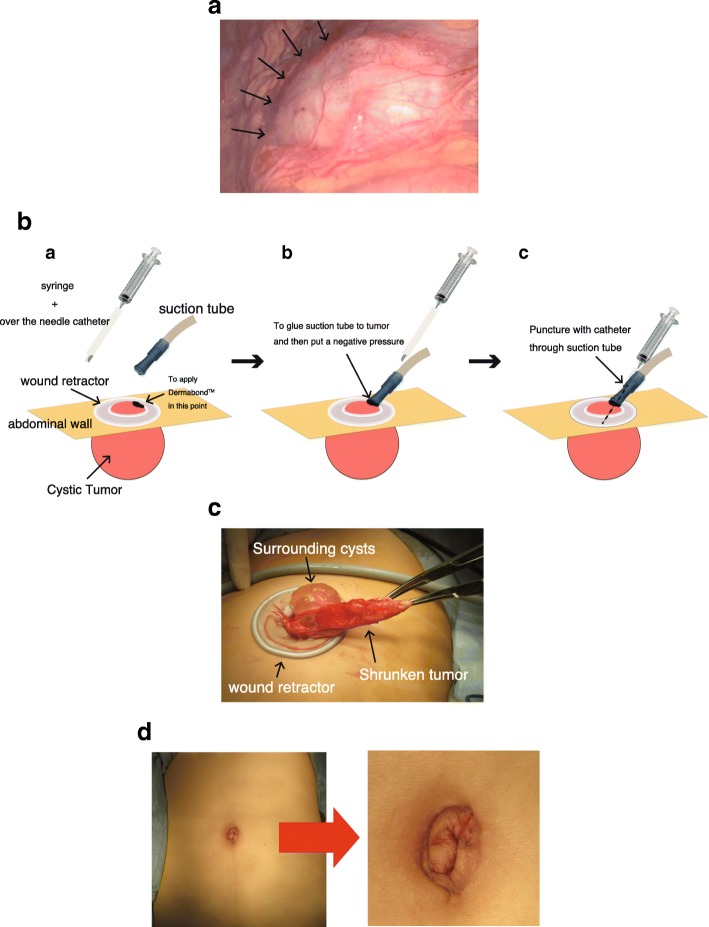
Surgical photographs. **a** A large cyst derived from the greater omentum, detected during laparoscopic surgery (arrows). **b** Schematic illustration of the operative procedure (a–c). The cystic tumor was punctured using a 16-gage over-the-needle catheter via a plastic suction tube, and the fluid was aspirated from the cyst. The glue and suction tube with negative pressure prevented spillage of any cyst fluid into the abdominal cavity. **c** Retraction of the large cyst after the aspiration of the cyst fluid. **d** The postoperative wound. The operation was successfully completed with only a small wound

**Fig. 3:**
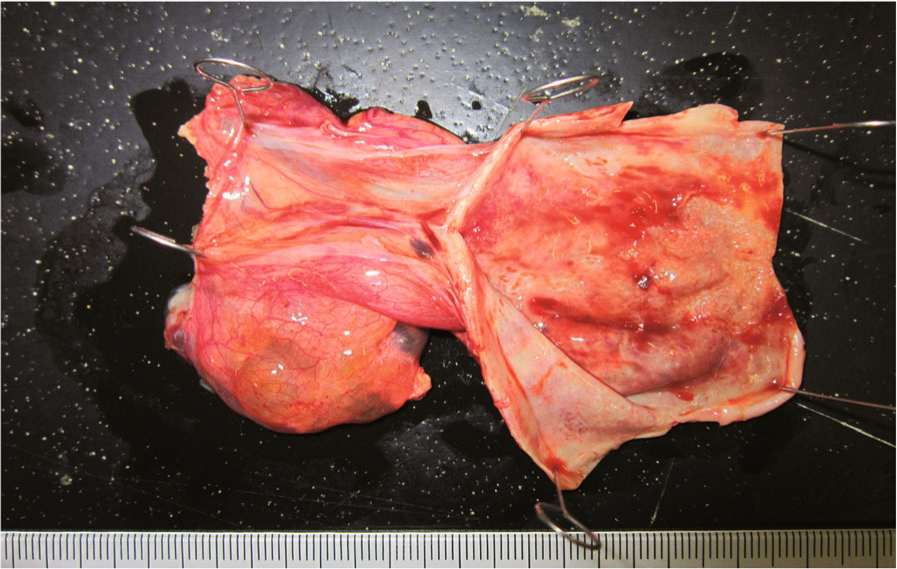
Resection specimen. The diagnosis was cystic lymphangioma, and the histopathological findings showed the cystic tissue to be benign. The cytology (cyst fluid) was class II

A single-incision access platform and wound protector were introduced through a 1.5-cm transumbilical skin incision. Laparoscopy showed a large cyst derived from the greater omentum (Fig. 2a), which was moved to a position under the umbilical wound. The cyst fluid (which was serous in nature) was aspirated using a tissue adhesive (Dermabond™, Ethicon Inc., Somerville, NJ), a suction tube with negative pressure, and a 16-gage over-the-needle catheter and syringe; this reduced the size of the tumor and none of the cyst fluid was released into the abdominal cavity (Fig. 2b, c). Then, the tumor was successfully removed via the small incision (Fig. 2c, d) and was diagnosed histopathologically as a cystic lymphangioma (Fig. 3).

The surgery was uneventful, and the postoperative recovery was normal.

### Discussion

Intraperitoneal cystic tumors are rare. A large cystic tumor usually requires a large skin incision for its removal. However, if the size of the cyst can be reduced by extracting its contents, it is possible to operate via a small wound [8]. In the present case, an abdominal cystic tumor was successfully removed via a small incision after aspirating the cyst fluid and thereby reducing the size of the tumor. The cystic fluid was successfully aspirated without spillage using a tissue adhesive, suction tube, and 16-gage over-the-needle catheter. The abdominal laparoscopic procedure used a smaller incision than is required for conventional laparotomy, and the laparoscope allowed more detailed observation, which was useful for the operative diagnosis.

Tumors close to the body surface may be resected via a small incision without a laparoscope, but movable tumors that shift to a site distant from the body surface cannot be resected in this way. However, even in such cases, a laparoscope can confirm the lesion and be used to guide it to an appropriate position. Even tumors fixed to a site distant from the body surface can be brought to the surface using a laparoscope after removing the surrounding tumor tissue. Laparoscopy allows for the manipulation of various types of tumor.

When cystic lesions can be reduced by aspirating the cyst contents, it is possible to perform a minimally invasive procedure with a small incision. However, it is crucial that none of the cyst contents leak into the peritoneal cavity. Preoperatively, our patient’s cystic tumor was predicted to be benign; therefore, surgery without lymph node dissection or extended resection was planned. However, the cyst contents may have been infected. In addition, a malignant cystic tumor could not be ruled out. Thus, care was taken to avoid any leakage of the cyst fluid into the surrounding areas. Trocar site recurrences due to bile leakage into the site of incision have been reported during cholecystectomy [9].

In our case, surgery was performed without any leakage of cyst fluid using medical equipment that is readily available at all surgical facilities. Laparoscopic-assisted surgery should be used to resect intraperitoneal cystic lesions wherever possible. This method can be applied to various types of cases, such as gynecological abdominal cystic diseases [7, 10]. The procedure used for the present case was easy to perform and required no special materials.

## Conclusions

Here, we described the case of a young woman who underwent laparoscopic-assisted surgery for a large abdominal cystic lymphangioma. The procedure can be easy to perform and is particularly beneficial for young women with various abdominal cystic lesions.

## Abbreviations

CT: Computed tomography
MRI: Magnetic resonance imaging
